# Laparoscopic low anterior resection for rectal cancer wherein the inferior mesenteric artery arose from the superior mesenteric artery: a case report

**DOI:** 10.1186/s40792-021-01254-z

**Published:** 2021-08-11

**Authors:** Takahiro Korai, Kenji Okita, Toshihiko Nishidate, Koichi Okuya, Emi Akizuki, Yu Sato, Atsushi Hamabe, Daisuke Kyuno, Masayuki Ishii, Ryo Miura, Masafumi Imamura, Minoru Nagayama, Takeshi Murakami, Takayuki Nobuoka, Tatsuya Ito, Ichiro Takemasa

**Affiliations:** Department of Surgery, Surgical Oncology and Science, Sapporo Medical University School of Medicine, 291 Minami-1-jo Nishi 16-chome, Chuo-ku, Sapporo, Hokkaido 060-8543 Japan

**Keywords:** Rectal cancer, Inferior mesenteric artery, Superior mesenteric artery

## Abstract

**Background:**

Few cases have been reported of colorectal cancer with inferior mesenteric artery (IMA) branching abnormalities; therefore, the lymphatic flow in such cases remains unknown. We report the first case of locally advanced rectal cancer in which the IMA arose from the superior mesenteric artery (SMA) in which we achieved to visualize the lymphatic flow.

**Case presentation:**

A 65-year-old woman complaining of bloody stools was investigated in our hospital and suspected with rectal cancer. Colonoscopy and abdominal enhanced computed tomography (CT) revealed a circumscribed, localized ulcerative tumor in the rectum. 3-Dimensional contrast-enhanced computed tomography (3D-CT) showed that the IMA arose from the SMA. The patient was diagnosed with rectal cancer (cT3N0M0, cStage IIa) and laparoscopic low anterior resection was performed. The sigmoid colon was resected using the medial approach. Only the plexus of the colic branch of the lumbar splanchnic nerve was observed at the site where the root of the IMA usually exists and showed interruption of the indocyanine green (ICG) fluorescence-illuminated lymphatics. The root of the IMA was ligated, and Japanese D3 lymphadenectomy was performed, preserving the accessory middle colic artery. All fluorescent lymph nodes were resected. The pathological diagnosis was pT4aN1aM0 stage IIIb. The patient’s postoperative course was uneventful. Adjuvant chemotherapy was administered, and the patient was recurrence-free at 1.5 years after surgery.

**Conclusions:**

We were able to perform safe and appropriate surgery oncologically, despite abnormal vascular anatomy, due to preoperative identification using 3D-CT and intraoperative navigation using ICG administration.

## Background

Japanese D3 lymphadenectomy is a standard treatment with respect to the extent of lymphadenectomy performed for advanced colorectal cancer in Japan. Similarly, complete mesocolic excision (CME) with central vascular ligation (CVL) is established in Europe. Both are achieved by ligating the root of the inferior mesenteric artery (IMA), for example left-sided advanced colorectal cancer [1, 2]. There have been few reports of colorectal cancer with IMA branching abnormalities, and to the best of our knowledge, no report exists describing the lymphatic flow in such cases [3–5].

We report the first case of locally advanced rectal cancer in which the IMA arose from the superior mesenteric artery (SMA) diagnosed by preoperative 3-dimensional contrast-enhanced computed tomography (3D-CT), and visualization of the lymphatic flow was achieved by the indocyanine green (ICG) navigation.

## Case presentation

**Fig. 1:**
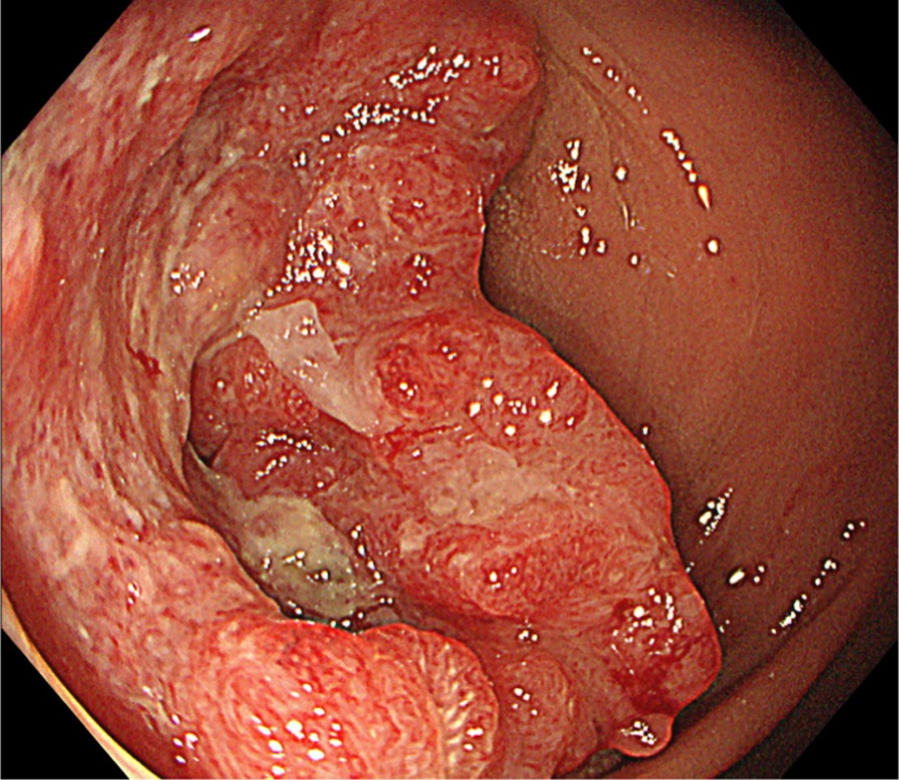
Preoperative colonoscopy examination. Colonoscopy shows a type 2 tumor, which is a circumscribed, localized, ulcerative tumor in the rectum

**Fig. 2:**
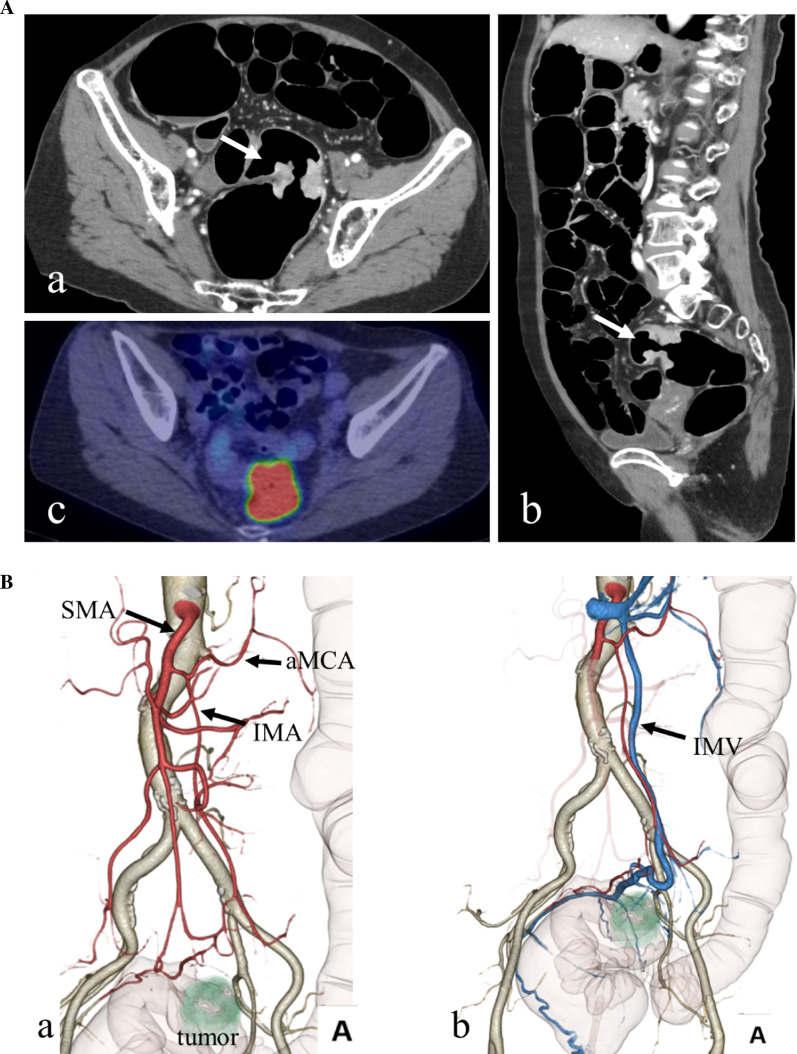
Preoperative contrast-enhanced computed tomography (CT) and PET–CT. **A** CT revealed wall thickening in the rectum (arrow). There were no enlarged lymph nodes or distant metastases (a: axial, b: sagittal). Positron emission tomography CT (PET–CT) revealed abnormal accumulation of FDG at the rectal tumor (c). **B** Three-dimensional-CT angiography reveals the IMA arising from the SMA (a). The IMA is accompanied by the IMV (b). (*IMA* inferior mesenteric artery, *SMA* superior mesenteric artery, *IMV* inferior mesenteric vein, *FDG* fluorodeoxyglucose

**Fig. 3:**
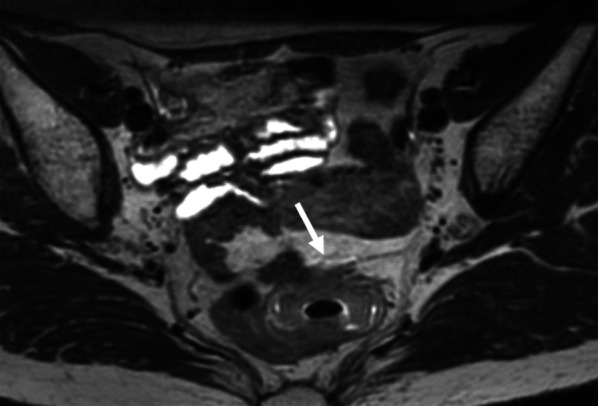
Preoperative magnetic resonance imaging (MRI). MRI revealed the tumor had invaded beyond the muscular layer of the rectum (arrow)

A 65-year-old woman presented with a complaint of bloody stools. The attending physician suspected a diagnosis of rectal cancer, and the patient was referred to our hospital for further investigation. On physical examination, her abdomen was soft and no mass was felt during palpation. Laboratory data on admission showed that the levels of tumor markers in the blood, including carcinoembryonic antigen and carbohydrate antigen 19-9, were within normal ranges. Colonoscopy revealed a circumscribed, localized, ulcerative tumor, 9 cm from the anal verge and 7 cm from the dentate line (Fig. 1). Abdominal enhanced computed tomography (CT) revealed wall thickening in the rectum and a high standardized uptake value max (SUVmax) of 21.3 (Fig. 2A). No enlarged lymph nodes or distant metastases were observed. On 3D-CT, the IMA was observed to originate from the SMA and was accompanied by the inferior mesenteric vein (IMV) (Fig. 2B). The line through which Japanese D3 lymphadenectomy would be performed was decided before the surgery (Fig. 2B). Magnetic resonance imaging (MRI) revealed tumor invasion beyond the muscular layer of the rectum (Fig. 3). We diagnosed the patient with rectal cancer (cT3N0M0, cStage IIa), and planned treatment with laparoscopic low anterior resection.

### Preoperative ICG local administration

This case was enrolled in the following studies which aimed to investigate the usefulness and limitations of treatment by laparoscopic colorectal surgery using the detection rate of ICG fluorescence markings with near-infrared light [6]. This study was approved by the Institutional Review Board of Sapporo Medical University, and informed consent was obtained from the patient. This clinical trial was registered in the University Hospital Medical Information Network (UMIN) Center (ID: UMIN000038982).

In this patient, the ICG solution was administered into the submucosal layer at two points around the tumor using a colonoscope 6 days before the laparoscopic colorectal surgery. Using a 26-gauge needle, 0.2 ml of normal saline solution was injected into the submucosal layer to form a submucosal elevation at two sites on the colonic wall opposite the tumor. Subsequently, 0.1 ml (ICG dose: 0.5 mg) of an ICG solution was injected into the elevated areas [6]. During the laparoscopic operation, we detected ICG fluorescence by using a laparoscopic near-infrared camera system (1688 AIM laparoscope; Stryker, San Jose, CA, USA).

### Surgical procedures

**Fig. 4:**
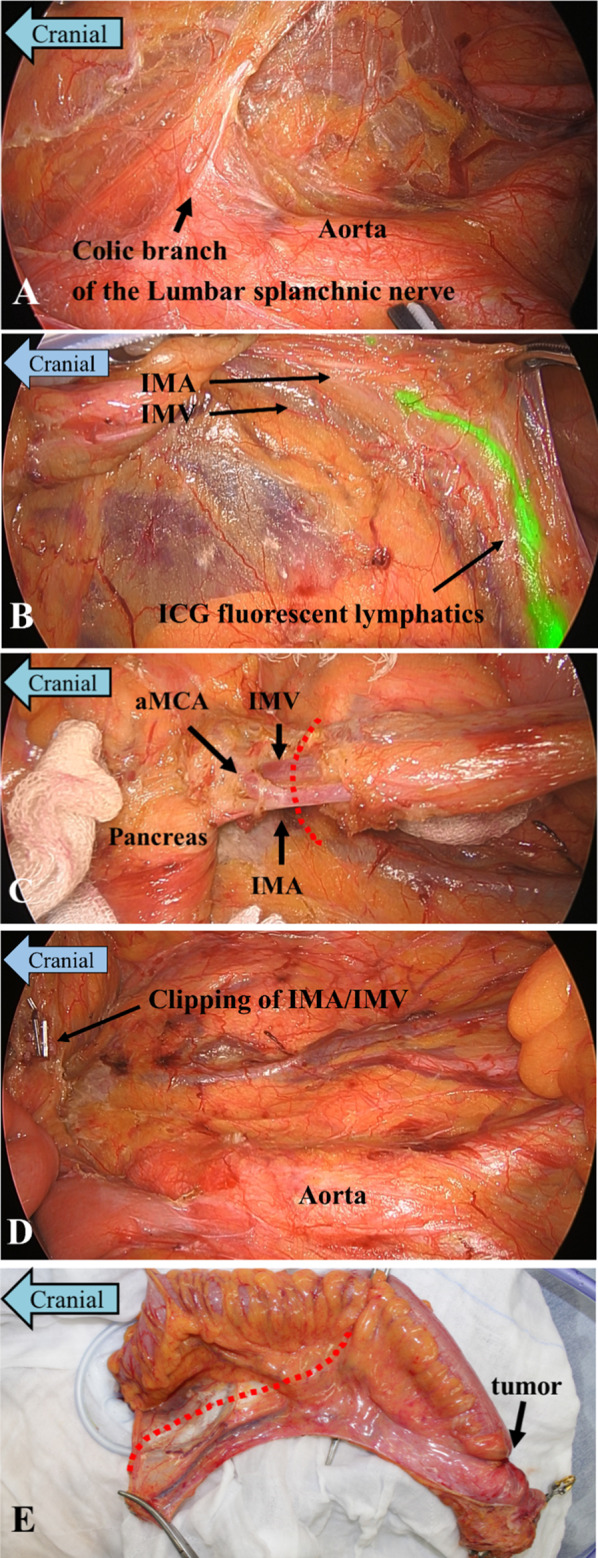
Intra-operative findings during laparoscopic low anterior resection. **A** At the usual site of the root of the IMA, IMA root was absent and only the colic branch of the lumbar splanchnic nerve was identified. **B** The ICG fluorescence-illuminated lymphatics were interrupted near where the IMA usually branches, and there was no central fluorescence-illuminated lymphatics. **C** We preserved the aMCA and ligated the IMA branching and the IMV on the same level, which is the upper border of the lymphadenectomy (dotted line). **D** All of the ICG fluorescence-illuminated lymphatics was resected. **E** The line of dissection of the mesentery were determined (dotted line). (*IMA* inferior mesenteric artery, *ICG* indocyanine green, *aMCA* accessory middle colic artery, *IMV* inferior mesenteric vein)

A small incision was made in the umbilical region, and surgery was initiated using a 5-port approach. The intra-abdominal cavity was observed, and no liver metastases or peritoneal dissemination were observed. The sigmoid colon was resected using the medial approach. The root of the IMA was not present at abdominal aorta; instead, at this site, we observed the presence of a plexus of the colic branch of the lumbar splanchnic nerve (Fig. 4A). The ICG fluorescence-illuminated lymphatics were interrupted near the root of the IMA, and no fluorescence was observed in the central region (Fig. 4B). The IMA did not originate from the abdominal aorta but from the accessory middle colic artery (aMCA), a branch of the SMA (Fig. 4C). We identified the IMA dorsally through the descending colon mesentery. By following it cranially, we were able to identify the branch of the aMCA and the root of the IMA. The IMA was ligated, and Japanese D3 lymphadenectomy was performed to preserve the aMCA at the level of the inferior border of the pancreas. An IMV was observed on the left side of the IMA in the mesentery of the descending colon, which was ligated next to the root of the IMA. All the fluorescence-illuminated lymph nodes were resected (Fig. 4D). After rectal evacuation and dissection, the intestine was guided out of the body, the margins of the oral and anal sides of the tumor were secured, the intestine was ligated, and the specimen was removed (Fig. 4E). ICG fluorescence imaging confirmed that blood flow resumed in the rest of the intestine 15 s after the intravenous administration of ICG and that there were no areas with poor blood flow, and the intestine was anastomosed using the double-stapling technique. After confirming that there were no leaks from the anastomotic site, the wound was closed. The operation lasted 220 min, and the estimated blood loss was 5 ml. No intraoperative blood transfusion was required.

### Macroscopic and pathological findings of the resected specimen

**Fig. 5:**
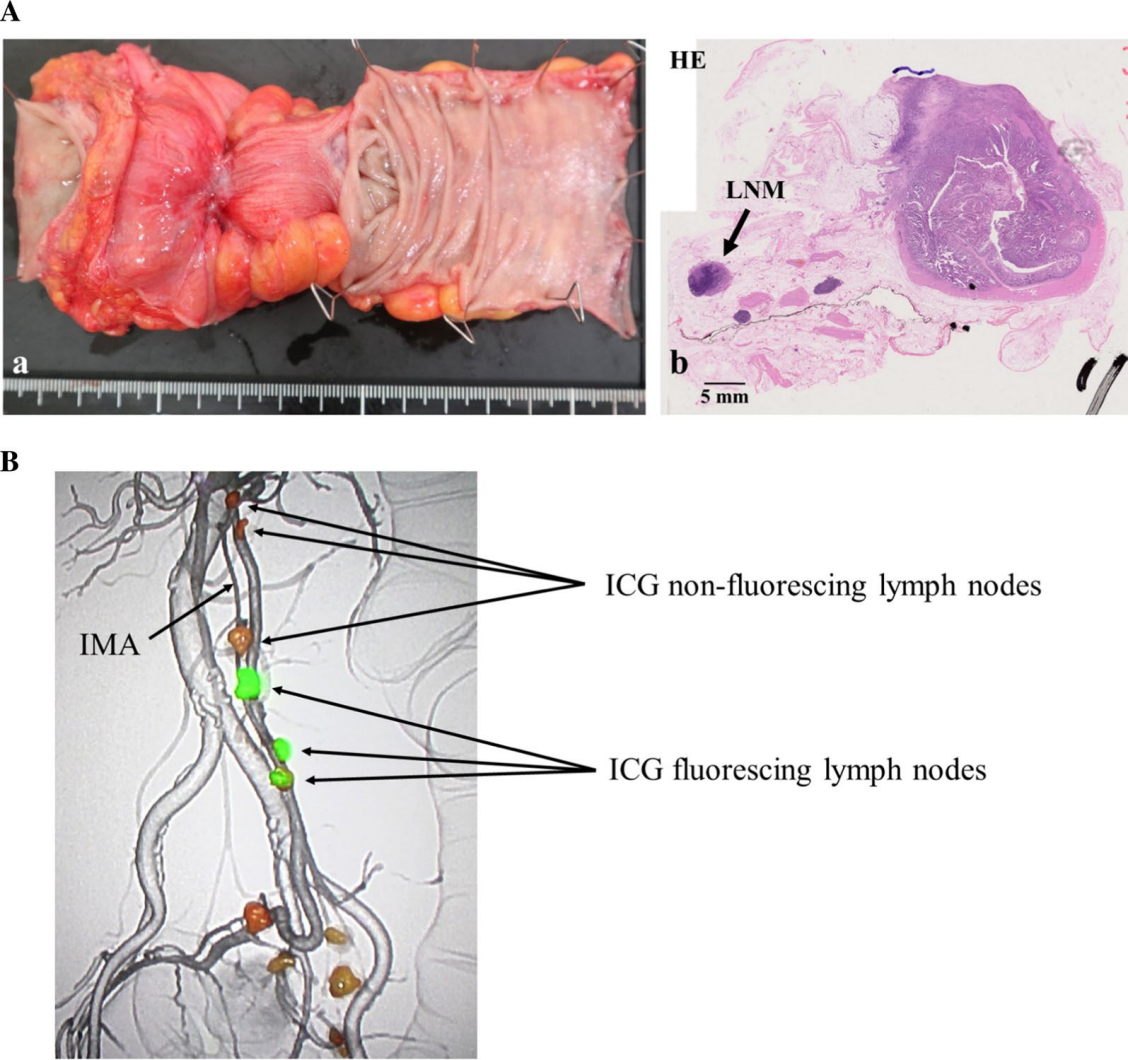
Gross and microscopic findings of the resected specimen. **A** Macroscopic findings of the resected specimen which was sliced transverse semi-opened for CRM measurements show serous membrane involvement (a). The pathological diagnosis was pT4aN1aM0 pStage IIIb (b). (*CRM* circumferential resection margin, *LNM* lymph node metastasis, *HE* hematoxylin and eosin). **B** Some ICG fluorescing lymph nodes which were detected up to the point where the IMA usually branches when a laparoscopic near-infrared camera system was used. There was no ICG fluorescing lymph nodes centrally (*ICG* indocyanine green, *IMA* inferior mesenteric artery)

Our department at the Sapporo Medical University is a part of a multi-institutional prospective observational study to validate the quality of pathological diagnosis, especially circumferential resection margin (CRM), by transverse slicing of semi-opened rectal specimens (UMIN000031735), and this case was included in this study. The tumor diameter was 80 mm × 30 mm. The pathological diagnosis was pT4aN1aM0 pStage IIIb because of serous membrane involvement of in the anterior wall and the presence of a positive lymph node (1/26 lymph nodes) (Fig. 5A). The CRM was 4 mm. ICG-fluoresced lymph nodes were observed anterior to the abdominal aorta, near a plexus of the colic branch of the lumbar splanchnic nerve, and there were no ICG-fluoresced lymph nodes at the position the root of the IMA (Fig. 5B).

### Postoperative course

The postoperative course was uneventful, and the patient was discharged on the 10th postoperative day. Eight courses of postoperative adjuvant chemotherapy (XELOX; Xeloda + oxaliplatin) were administered, and the patient was recurrence-free 1.5 years after surgery.

## Discussion

Variations or anomalies in the IMA anatomy are rare. In the report by Kakihara et al., abdominal angiography in 3182 patients revealed occasional IMA bifurcation anomalies, consisting of a defect in one patient (0.03%), running anomaly due to visceral retroversion in two patients (0.06%), and bifurcation from the root of the SMA in two patients (0.06%) [5]. Similarly, studies by Kondo et al. and Minamizawa et al. reported presence of abnormality, in which IMA branched from SMA, in 0.07% (one among 1516 cases) and 0.05% of the primary resection cases of colorectal cancer, respectively [7, 8].

**Fig. 6:**
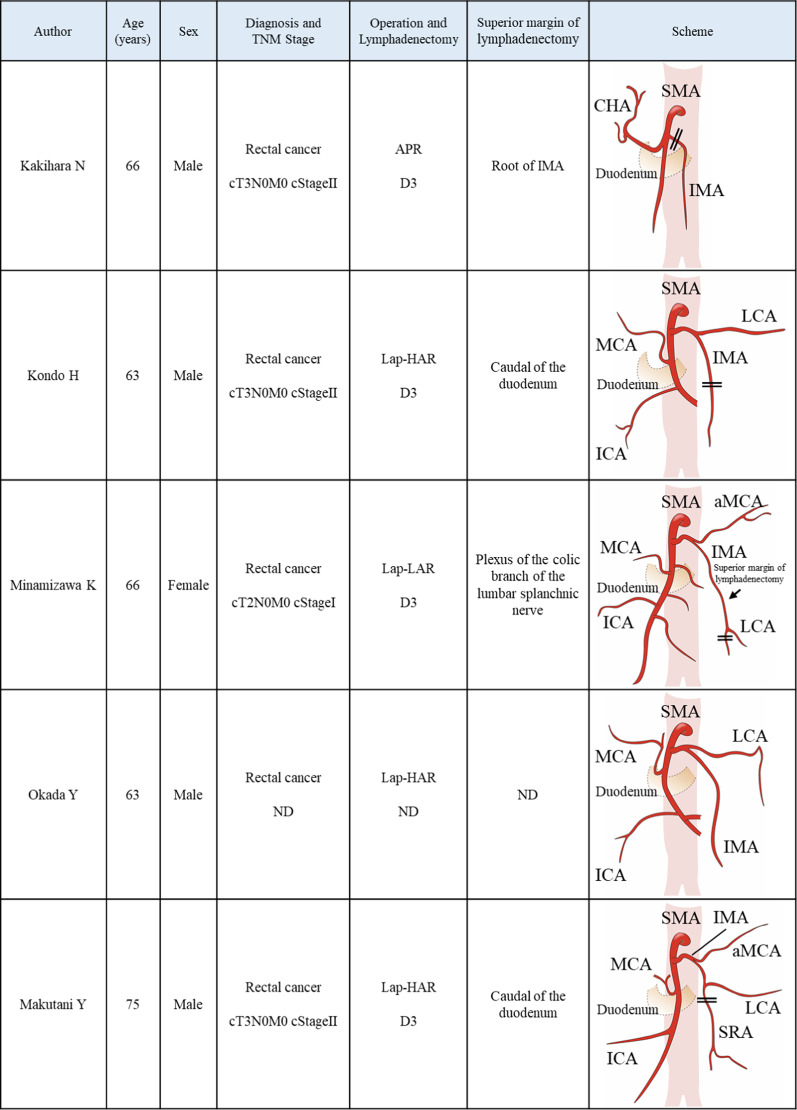
Reported cases of colorectal cancer with abnormal branching of the inferior mesenteric artery. In most cases, the superior margin of lymphadenectomy was set at the inferior border of the duodenum at the position where the IMA normally originates from the abdominal aorta or at the plexus of the colic branch of the lumbar splanchnic nerve. (*TNM Stage* 8th edition of the AJCC-TNM Staging, *c* clinical, *APR* abdominoperineal resection, *Lap-HAR* laparoscopic high anterior resection, *Lap-LAR* laparoscopic low anterior resection, *ND* not described, *D3* Japanese D3 lymphadenectomy, *SMA* superior mesenteric artery, *IMA* inferior mesenteric artery, *CHA* common hepatic artery, *LCA* left colic artery, *MCA* middle colic artery, *ICA* ileocolic artery, *aMCA* accessory middle colic artery, *SRA* superior rectal artery)

Using “variation” and “inferior mesenteric artery” as search keywords, we identified six papers describing anatomical abnormal branching of the root of IMA in autopsy cases reported on PubMed from 1968 to 2021 [9–14]. However, we found only case report in the English literature for colorectal cancer cases wherein IMA arose from the SMA. Okada et al. focused his report on the arterial anatomy, therefore, the details of the surgery for rectal cancer were unknown [14]. Using a Japanese literature search in “Igaku Chuo Zasshi” (Central Journal of Medicine), we found five cases, including our case (Fig. 6) [5, 7, 8, 14, 15]. In most cases, the superior margin of lymphadenectomy was set at the inferior border of the duodenum at the position where the IMA normally originates from the abdominal aorta or at the plexus of the colic branch of the lumbar splanchnic nerve. In this case, as in the case reported by Kakihara et al., Japanese D3 lymphadenectomy was performed by ligating the root of the IMA.

It has also been reported that preoperative 3D-CT is informative and helpful for surgeons to assess the vascular anatomy, especially the vascular variability around the SMA [14, 16, 17]. Bifurcation abnormalities are uncommon with IMA, however, preoperative confirmation of anatomy of IMA using 3D-CT is useful information for surgeons in left-sided colorectal cancer surgery. In our department, 3D-CT is performed in all cases of colorectal cancer; therefore, bifurcation abnormality of the IMA could be identified preoperatively in this case, and the surgery could be performed safely without intraoperative complications such as vascular injury.

ICG fluorescence imaging has been widely used to evaluate the blood and lymphatic flow for colorectal cancer surgery [18–21]. The mechanism of sometimes selective illumination of lymph nodes is unclear [22]; however, in the present case, the ICG-fluorescence-illuminated lymphatic flow was observed to end at the position where the IMA normally originates from the abdominal aorta. In this case, the application of the latest equipment of a laparoscopic near-infrared camera system helped us perform complete CME as well as Japanese D3 lymphadenectomy.

Tandler [23, 24] reported that during arterial development of abdominal organs in the fetus measuring 5 mm, several primitive mesenteric arteries arise segmentally from the dorsal aorta and supply their respective primordia. In the early stages of development, longitudinal anastomoses connect the arteries vertically in the dorsal mesentery [23, 24]. However, if abnormal partial disappearance of the primitive mesenteric artery and longitudinal anastomosis occurs, branching abnormalities such as that seen in the present case may occur. The arc of Riolan, a vessel assumed to connect the proximal segments of the MCA and left colic artery has an incidence of 8–10% [25]. It is not clear if this longitudinal anastomosis connecting the SMA and IMA is the same as the arc of Riolan; however, we believe that the bifurcation abnormality of the IMA in this case is unrelated to the arc of Riolan based on the vascular morphology. In this case, only the colic branch from the lumbar splanchnic nerve was found in the position where the IMA normally branches from the abdominal aorta, suggesting that the disappearance of the primitive mesenteric artery of the hindgut during development may have caused the abnormal IMA bifurcation, as discussed by Tandler.

Based on the above, we suspected that the IMA originated from the abdominal aorta and was accompanied by lymphatics during fetal life; however, the root of the IMA disappeared during the developmental process, and lymphatics ascending along the IMA persisted even after the disappearance of the root of the IMA and did not get redirected towards the SMA. Although the extent of lymphadenectomy is not established in such cases, ligation at the bifurcation of IMA was considered to be oncologically appropriate in this case. Since surgical cases of colorectal cancer with abnormal branching of the IMA are rare, further studies are required to validate these findings.

## Conclusions

We reported the first case of locally advanced rectal cancer with abnormal branching of the IMA from the SMA in whom safe and appropriate surgery was performed oncologically due to preoperative identification using 3D-CT and intraoperative navigation using ICG administration.

## Data Availability

The dataset supporting the conclusions of this article is included within the article.

## Abbreviations

CME: Complete mesocolic excision
CVL: Central vascular ligation
IMA: Inferior mesenteric artery
SMA: Superior mesenteric artery
CT: Computed tomography
SUVmax: Standardized uptake value max
IMV: Inferior mesenteric vein
3D-CT: 3-Dimensional contrast-enhanced computed tomography
MRI: Magnetic resonance imaging
ICG: Indocyanine green
aMCA: Accessory middle colic artery
CRM: Circumferential resection margin
LCA: Left colic artery
